# Short-term apparent brain tissue changes are contributed by cerebral blood flow alterations

**DOI:** 10.1371/journal.pone.0182182

**Published:** 2017-08-18

**Authors:** Qiu Ge, Wei Peng, Jian Zhang, Xuchu Weng, Yong Zhang, Thomas Liu, Yu-Feng Zang, Ze Wang

**Affiliations:** 1 Center for Cognition and Brain Disorders, Department of Psychology, Hangzhou Normal University, Hangzhou, China; 2 Department of Physics, Hangzhou Normal University, Hangzhou, China; 3 GE Healthcare Beijing, China; 4 Department of Radiology, University of California San Diego, San Diego, United States of America; 5 Department of Radiology, Lewis Katz School of Medicine, Temple University, Philadelphia, United States of America; Duke-NUS Graduate Medical School, SINGAPORE

## Abstract

Structural MRI (sMRI)-identified tissue “growth” after neuropsychological training has been reported in many studies but the origins of those apparent tissue changes (ATC) still remain elusive. One possible contributor to ATC is brain perfusion since T1-weighted MRI, the tool used to identify ATC, is sensitive to perfusion-change induced tissue T1 alterations. To test the hypothetical perfusion contribution to ATC, sMRI data were acquired before and after short-term global and regional perfusion manipulations via intaking a 200 mg caffeine pill and performing a sensorimotor task. Caffeine intake caused a global CBF reduction and apparent tissue density reduction in temporal cortex, anterior cingulate cortex, and the limbic area; sensorimotor task induced CBF increase and apparent tissue increase in spatially overlapped brain regions. After compensating CBF alterations through a voxel-wise regression, the ATC patterns demonstrated in both experiments were substantially suppressed. These data clearly proved existence of the perfusion contribution to short-term ATC, and suggested a need for correcting perfusion changes in longitudinal T1-weighted structural MRI analysis if a short-term design is used.

## Introduction

T1-weighted structural MRI (sMRI) is a major MRI tool for study brain structures due to the high spatial resolution, signal-to-noise-ratio, and the high intra-tissue signal contrast that it can provide. Using sMRI, researchers have identified apparent tissue changes (ATC) after neuropsychological training or medication [1–13]. While those findings might provide empirical evidence of brain plasticity, their underlying neuro-mechanism still remains unclear [13, 14] especially when there is no solid evidence of short-term neurogenesis, synaptogenesis, or gliogenesis in adult human brain [15–18]. Because functional brain changes often are accompanied by perfusion change [19], and nearly all aforementioned short-term or long-term learning or training brain plasticity studies showed improved brain functions, one possible but still not fully proven non-structural source of ATC could be brain perfusion. Because arterial blood has larger T1 than brain tissues[20], substantial perfusion alterations would subsequently change tissue T1 and induce ATC in the T1 image based brain structure analysis using tools like the voxel-based morphometry (VBM)[21]. Perfusion changes can also change tissue volume because of the changed hydration. These postulations however have thus far only been evidenced in one paper. In a study assessing the effects of GABAnergic medicine, Baclofen on blocking the smoking-cue induced brain activations in chronic smokers, Franklin et al. [22] examined both brain tissue changes and CBF changes after chronic smokers took one-dose of Baclofen. They found partially overlapped baseline CBF reduction and ATC patterns. Probably due to the limited sample size and or the non-specific CBF effects of the one-dose medicine (Baclofen), the CBF reductions in that study were defined with a modest significance level. In addition, the data were acquired from a 3-week smoking cessation trial, which can be subject to various confounds including nicotine effects, time, other drug use, etc, making it non-ideal for delineating CBF effects from the observed ATC. For example, abstinence from nicotine smoking can both increase and decrease regional blood flow [23, 24]. Smokers may often drink alcohol, which affects blood vessels and susbsequently blood flow too.

The purpose of this paper was to verify the hypothetical CBF contributions to short-term ATC in normal adult brain using two established CBF manipulation paradigms: caffeine ingestion and visual-sensorimotor functional activation. We focus on very-short term ATC because it is nearly impossible to have real tissue alterations in the normal brain after taking low-dose caffeine and performing a short sensorimotor task. As compared to previous work[22], several novelties were presented in this study. First, the two approaches are known to have reliable CBF effects. Acute caffeine intake is known to reduce global CBF [25–30] and visual and sensorimotor function activation is a classical fMRI paradigm known to increase regional CBF in the visual and sensorimotor network. Using these two approaches, we can see the effects of both global and regional CBF-induced apparent tissue changes. Second, to avoid other confounds such as brain disorders, age, and time, we included young healthy subjects and used a short-time design. Third, imaging data were acquired and processed with updated techniques. Tissue changes were examined with the latest longitudinal VBM analysis method[21], which was recommended in the longitudinal VBM method comparison paper [31]. CBF was measured using pseudo-continuous arterial spin-labeled (pCASL) perfusion MRI sequence with background suppressed [32]. Such kind of sequences has been recently demonstrated to yield more significant CBF difference after caffeine ingestion [30].

## Materials and methods

### Subjects

This study was conducted at the Center for Cognition and Brain Disorders (CCBD) in Hangzhou Normal University. All procedures were approved by the CCBD Institutional Review Board (approval No. 20130819), and adhered to the Declaration of Helsinki. All subjects were right-handed and were recruited from Hangzhou Normal University and local community in Hangzhou, China from Oct 2013 to Apr 2015. They provided signed consent forms prior to entry into the study. Subject exclusion criteria were: abnormal structural MRI, a history of head trauma or other injury resulting in loss of consciousness lasting greater than three minutes or associated with skull fracture or inter-cranial bleeding, with any embedded brain or body stimulation devices, with any ferromagnetic or magnetic susceptible materials or objects on or within their body, having any neuropsychological problems as defined by the Mini-International Neuropsychiatric Interview (MINI)[33], taking any medications that can affect CBF in the past 10 days. Additional exclusion criteria were used for the subjects recruited for the caffeine intake experiment: allergic to caffeine, drinking more than 1 cup of coffee or tea in the past week and more than 10 cups of coffee or tea in the past half year.

50 young healthy Han people (25 males and 25 females, age: 22.76±2.22 years (mean and standard deviation (STD)), age range: 19~27 years) who had not drunken more than 1 cup of coffee and 1 cup of tea in the past week participated in the caffeine experiment. The sensorimotor task fMRI included 20 young health Han subjects (10 females, 10 males, age: 23.1±2.68 years, range19-28). No other demographic data were collected.

### Design

In the caffeine experiment, all participants attended two imaging scan sessions in two days with exactly 24 hours apart during the daytime between 9am and 5pm. No coffee or caffeine contained beverage or food were allowed since 24 hours before the first experiment. Caffeine has a mean serum half-life of 5.7 hours [34], and the 24 hour interval between the two scan days was chosen to allow a full digestion of caffeine if it was taken in the first scan day. The order of being caffeine free or taking caffeine prior to scan was counter-balanced with half of subjects took a caffeine pill (200 mg) in the first day. For both sessions, subjects were asked to rest for the same amount of time (30 minutes) outside of the scanner room before going inside the scanner. For the caffeine intake session, subjects took a caffeine pill with water and waited 30 minutes before going inside the scanner for MRI; for the non-caffeine session, subjects only took the same amount of water as in the caffeine intake session and waited 30 minutes before going inside the scanner. For each session, both a T1-weighted high resolution structural MRI and a six-minute brain perfusion MRI were acquired. Structural MRI lasted for 4 mins and 33 secs. ASL scan was 4 mins and 32 secs. The subjects were asked to stay awake while doing nothing during both scans. The sensorimotor fMRI experiment followed a block-wise design, consisted of two resting scan sessions (one for structural MRI and one for ASL MRI) and two task activation scan sessions (one for structural MRI and one for ASL MRI). During the resting session (4 mins and 33 secs when sMRI was acquired and 4 mins and 32 secs when ASL MRI was acquired), subjects were asked to keep eyes open and think about nothing for the entire scan time (either during the structural scan or ASL scan). During the task condition (4 mins and 33 secs when sMRI was acquired and 4 mins and 32 secs when ASL MRI was acquired), subjects were asked to watch an 8 Hz reversing black and white checkerboard and perform a self-paced two-hand fingertapping task for the entire scan time (either during the structural scan or ASL scan). The order of them within both the resting and task scan section was counterbalanced.

### Imaging parameters

Imaging experiments were performed on a 3.0T whole-body GE 750 MR scanner (GE, Milwaukee, US), using a standard 8-channel receive array. Structural images were acquired using a T1-weighted inversion prepared 3D spoiled gradient echo (IR-SPGR) sequence with FOV = 256x256mm^2^, inversion time = 450 ms, TR/TE = 7.2/2.1ms, 256x256 matrix, 176 sagittal slices with slice thickness = 1 mm. ASL scan was performed using a GE product pseudo continuous arterial spin labeling (PCASL) perfusion MRI sequence. Three interleaved images with and without labeling were obtained using a 3D fast-spin echo encoded spiral readout imaging sequence with 6 shots, 40 axial slices, TR = 4690ms, TE = 10.9ms, FOV = 220x220 mm2, slice thickness = 3.4, labeling time = 1.5s, post label delay = 1525ms, bandwidth = 62.50Khz, matrix = 512x512.

### Data processing

#### Longitudinal VBM

The longitudinal VBM algorithm [21] was used. A midterm (between pre- and post-caffeine ingestion, and between the rest and task fMRI conditions) structural image was generated for each subject using a fast diffeomorphic registration algorithm, an exponentiated Lie algebra algorithm (DARTEL)[35] implemented in SPM12. During the same process, the longitudinal (pre- minus post-caffeine, and task minus rest) structural brain difference was also created. The midterm individual brains were segmented into gray matter (GM), white matter (WM), cerebrospinal fluid (CSF) using the new segmentation algorithm in SPM12 (http://www.fil.ion.ucl.ac.uk/spm/). A brain template capturing the common features of all assessed subjects was then generated from the segmented midterm brains and was subsequently registered to the Montreal Neurological Institute (MNI) standard space. The longitudinal structural brain difference maps were eventually registered and resampled into the MNI space.

#### ASL data processing

CBF calculation was performed using an SPM-based ASL data processing toolbox [36]. The M0 map was registered to the midterm structural MRI generated as above. The same registration transform was then applied to the CBF map, which was subsequently registered into the MNI space using the aforementioned DARTEL created structural MRI based individual brain to MNI space transform. The pre- minus post-caffeine and task-rest CBF difference maps were calculated for the following analyses.

#### Statistical analysis

One sample t-tests were used to assess the caffeine induced and the task-activation induced tissue and CBF changes using the above mentioned tissue probability difference maps and CBF difference maps, respectively.

CBF difference was correlated to tissue difference at each voxel using simple regression for the caffeine experiment data and sensorimotor task experiment data separately.

CBF difference was regressed out from the tissue difference map at each voxel. One-sample t-test was conducted for the residual of the tissue difference maps to check whether controlling CBF changes would reduce the apparent tissue changes (caused by caffeine or task activation).

## Results

### CBF and grey matter changes due to caffeine ingestion

**Fig 1:**
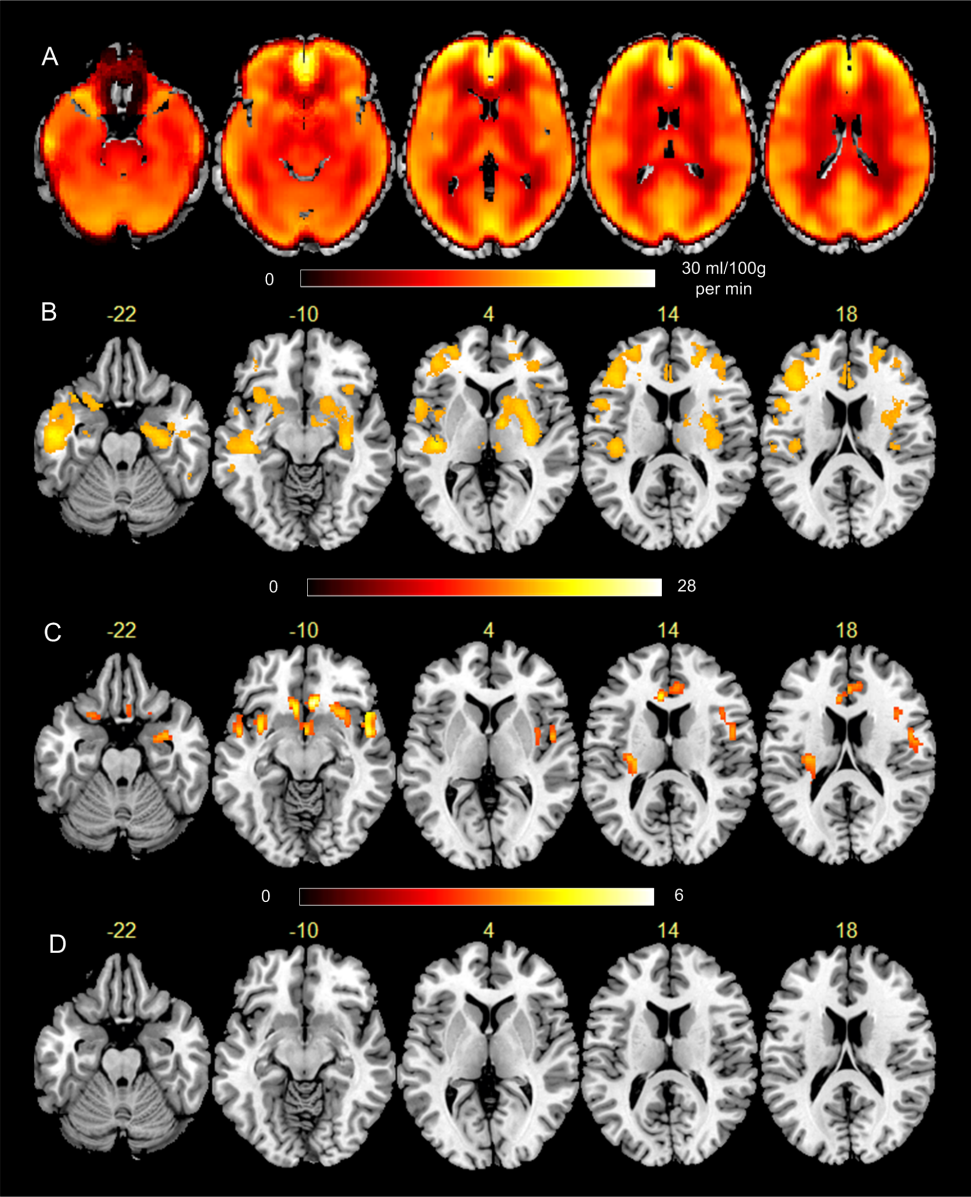
Statistical analysis results of the caffeine experiment (n = 50). A) Caffeine-induced mean CBF reduction (shown as the mean pre-caffeine minus post-caffeine CBF difference). The colorbar underneath the figure indicates the color window for displaying the CBF difference; B) T-map of the pre- minus post-caffeine CBF paired-t test thresholded at t> = 17. The colorbar under the pictures indicate the color window for the t values; C) caffeine-induced ATC (pre-caffeine minus post-caffeine). The colorbar under the pictures is the display window for the t-values; D) pre-post caffeine-intake ATC after controlling the pre-post CBF change. The number above each slice indicates the axial slice location in the MNI space.

Fig 1 shows the group level pre-caffeine minus post caffeine analysis results. Fig 1A is the mean CBF difference (pre-caffeine minus post caffeine). Caffeine ingestion (a 200mg pill) caused significant CBF reduction (p<0.005, cluster size<100, alpha<0.05 with Monte Carlo simulation-based multiple comparison correction provided by 3dClustSim (https://afni.nimh.nih.gov/pub/dist/doc/program_help/ 3dClustSim.html)) in the entire brain (Fig 1A). The mean CBF reduction in the entire brain was 15.3 ml/100g/min. Fig 1B is the paired-t test of the pre- and post-caffeine CBF difference thresholded at t> = 17 in order to show the peak locations. The extremely high threshold was chosen to only show the most prominent CBF difference so we could compare the patterns to those in VBM analysis. Fig 1C is the thresholded t map of the longitudinal VBM analysis. Under the same significance threshold, grey matter intensity reduction after taking caffeine was observed in hippocampus, orbito-frontal cortex, ventral striatum, anterior cingulate cortex (ACC), insula, and superior temporal cortex (Fig 1C). Fig 1D is the VBM analysis result after regressing out the pre-post caffeine CBF difference from the tissue density difference. No significant caffeine induced tissue density change was observed after regressing out CBF reductions at each voxel.

### CBF and grey matter changes due to sensorimotor task activation

**Fig 2:**
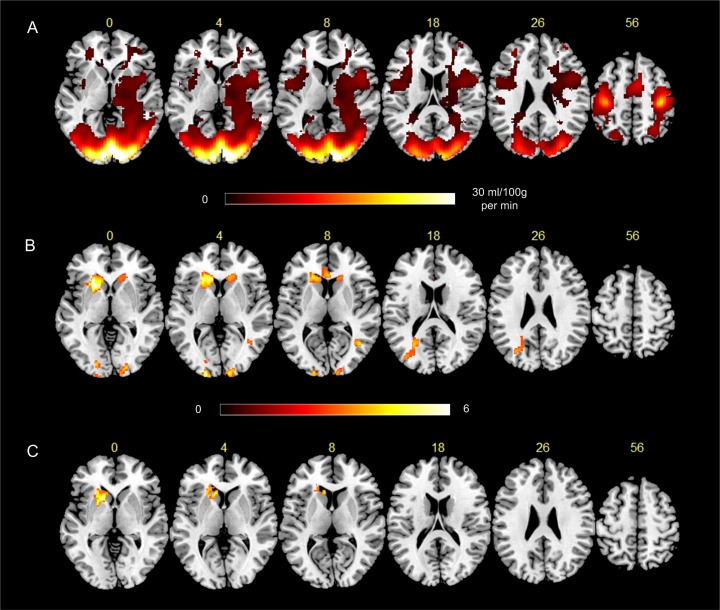
Statistical analysis results of the sensorimotor task fMRI experiment. A) Task-induced mean CBF reduction (shown as the mean pre- minus post-task CBF difference), B) task-induced ATC (pre-caffeine minus post-caffeine), C) pre-post task ATC after controlling the pre-post CBF change. The number above each slice indicates the slice location in the MNI space. The two colorbars indicate the display window for A and B: the one under A indicates the CBF range of the displayed mean CBF difference; under B indicates the parametric t-map of the one-sample t-test of the pre-post caffeine tissue volume change.

Fig 2 shows the results of the sensorimotor task performance experiment. Fig 2A is the statistically thresholded post-pre task CBF difference. Fig 2B is the thresholded t-map of the post-pre task tissue volume change analysis. Sensorimotor task activation caused significant (p<0.005, cluster size>100, alpha<0.05 with Monte Carlo simulation-based multiple comparison using 3dClustSim) CBF increase (hot color spots in Fig 2A) in the sensorimotor area (visual cortex, thalamus, putamen, insula, motor cortex, supplementary motor area) and prefrontal cortex. Using the same voxel-wise significance level, sensorimotor activation resulted in significant apparent tissue density increase (hot spots in Fig 2B) in bilateral basal ganglia and visual cortex. The apparent visual cortex tissue change clusters overlapped with task-induced CBF changes. In caudate, ATC partially overlapped with CBF changes. Fig 2C shows that regressing out CBF changes at each voxel suppressed the apparent gray matter increase pattern in visual cortex and greatly reduced ATC in caudate (the significance threshold was the same as used above).

## Discussion

We demonstrated that short-term CBF reduction or CBF increase induced apparent brain tissue decrease or increase, respectively in the T1-weighted MRI-based VBM analysis. Caffeine ingestion caused a global CBF reduction and focal apparent tissue intensity reduction. Visual sensorimotor task activation caused regional CBF increase and apparent tissue density increase. After controlling for CBF changes (regressing out CBF changes from the tissue difference), the apparent tissue density alterations were substantially suppressed. Since taking a small dose of caffeine for 30 minutes or performing a short-time visual sensorimotor task for 9 minutes are nearly impossible to result in genesis of neurons or non-neuronal cells at a massive level to be observerable in sMRI, our data reasonably evidenced that short-term sMRI-derived tissue changes are likely contributed by CBF alterations.

Previous learning-induced brain tissue increase studies noticed after-training functional improvement but didn't measure change of baseline CBF [1–13], which may explain why few studies discussed the possibility of CBF-change induced ATC. While we showed evidence of the non-structural contribution to the short-term sMRI-derived apparent tissue changes, our data can not be over-explained to exclude the existence of having real structural changes after long-time learning or training (such as months or years) or during the normal brain development [37, 38]. Regardlessly, those long-term morphology changes should be assessed using advanced MR imaging methods to be more readily interpretable [39]. Regarding the neuronal and non-neuronal cause debate, our data showed both ways. In the caffeine experiment, CBF reduction is mainly assumed to be induced by the neurovascular effects of caffeine. In other words, the CBF-induced ATC is caused by a non-neuronal CBF variation event. In the visual-sensorimotor task, CBF increase is caused by neuronal activation, meaning that the observed ATC was contributed to by neuronal events.

Caffeine induced a global CBF reduction but only caused GM reduction in a few brain regions. The spatial mismatch can be partly explained by the ATC-identification process in VBM. In VBM, only two types of brain changes can be captured. The first is the tissue volume difference which can be captured by the Jacobian during spatial brain normalization (into the standard brain space). The second is the tissue histograms are first created in order to segment the brain into different tissue type (such as grey matter, white matter, and CSF) with a certain probability. When CBF alterations change the contrast between different brain tissues, a change of sMRI signal intensity histogram will occur, and consequently introduce ATCs. The reason for having a global CBF reduction but a spatially restricted ATC may be that the global CBF reduction did not change brain tissue intensity contrast significantly in most of the brain, resulting in no change to the tissue intensity histograms. This may also explain why only a small portion of the visual cortex showed significant ATC but nearly the entire visual cortex presented CBF increase. Interestingly, basal ganglia (caudate and putamen) showed big ATC clusters with minor overlap with the CBF increase clusters. Such mismatch may be caused by the non-linear component of the CBF and sMRI relationship. Their non-linear relationship can be even perceived in the spatially overlapped regions. As shown in the supplementary figures, in the tissue change and CBF change overlapped brain areas, the caffeine-induced CBF changes didn’t show significant linear correlations to the tissue changes, though the task-activation induced CBF changes showed strong correlations to the task-activation induced tissue changes. This non-linear relationship also suggests existence of other contributing factors. For example, brain metabolite concentration changes in response to functional activation in a regionally inhomogeneous way. Because metabolites have different T1 than tissue, their concentration changes could induce ATC as well. In normal healthy condition or when the impaired brain state is recovering toward the healthy condition, increased brain activity induced an increase of blood flow through the CBF-metabolism coupling though the coupling ratio may vary by caffeine [40, 41] or aging [42]. Nevertheless, regressing out CBF changes at each voxel substantially suppressed ATC in both experiments. The observed concurrent CBF change and ATC as well as the regression CBF out VBM analysis results suggest the hypothetical short-term CBF change-induced ATC and the need for controlling CBF during VBM analysis.

One concern might be raised is the head motion difference between the caffeine-free and caffeine sessions or between the baseline and sensorimotor task performance conditions. Motion surely is a concern for all MRI experiments and there is no technique available to completely avoid that. In our data, we didn’t notice motion artifacts in both sMRI and ASL MRI. In additionally acquired resting state fMRI, we explicitly estimated head motions using the 6 direction rigid body affine transformation based motion correction algorithm implemented in SPM. For the subjects included in this study, there were no significant motion differences between the two scan sessions. The ASL MRI was based on a spiral sampling trajectory. Repeatedly sampling the k-space center therein reduces sensitivity to motions. Longitudinal data analysis has been an active research topic and advanced analysis methods have been proposed for more than 2 data points without strong data property assumptions [43, 44]. In this study, we only acquired data at two timepoints, which still fits with the standard pair-wise statistical comparison model. Certainly, if more timepoints will be acquired in future study, the otherwise more complicated model should be used to analyze the data. It is very likely that other factors may contribute to the short-term tissue changes as well. Future studies should include more imaging modalities using other advanced MRI techniques to have a more comprehensive understanding of the short-term tissue changes or brain tissue plasticity [39]. We used caffeine and fingertapping task because the two experiment paradigms are known to induce CBF changes and are less likely to induce tissue changes, so we can explicitly verify whether CBF can cause tissue changes.

Several limitations should be mentioned. First, caffeine intake was not controlled with placebos. The CBF change results might be a result of both caffeine and placebo. However, this should not affect the verification of the major goal of this paper which was to verify the CBF contribution to ATC rather than caffeine or placebo per se. We chose this simple design because caffeine has been proven by many papers to induce robust CBF change. As no prior studies have reported a significant CBF reduction after taking placebo pills, the placebo effects if any should be a minor component to the identified CBF change. Second, CBF changes can be induced by metabolism change and cerebral blood volume. Metabolism change is usually much smaller than CBF and may not be detectable by sMRI-VBM. CBV is positively related to CBF given the mean transit time. If the blood vessel volume doesn’t change, CBV would have no effects on T1-weighted sMRI. But when increased CBV is accompanied by enlarged vessel volumes, it may induce decreased tissue volume to keep the total volume the same. In our data analysis, we didn’t find much difference between the analysis results with and without regional volume modulation (meaning keep or not keep the regional tissue volume unchanged after spatial brain normalization), suggesting that the effects of CBV are minor. On the other hand, tissue volume change due to vessel volume increase will relatively decrease the free water fraction of tissue and subsequently lead to a reduction of tissue T1[45]. That reduction would show up in sMRI-VBM as atrophy rather than “tissue growth”, which may in fact compensate part of the ATC. To further delineate CBV effects, CBV data should be collected in future studies.

In summary, we showed the non-structural contributions to the short-term sMRI-ATC by manipulating CBF in the normal brain. Our data suggest that controlling for CBF changes in sMRI data analysis should be necessary to delineate the real structural MRI findings at least for the short-term longitudinal studies. Certainly, our data did not exclude the previously observed long-term tissue changes though CBF contributions might still exist.

## Supporting information

S1 Fig The plot of caffeine induced tissue density reduction versus CBF reduction. Difference means pre-caffeine minus post-caffeine. Both measures were extracted from the suprathreshold sMRI-ATC regions shown in Fig 1B. No significant correlation between the caffeine-induced changes of the two measures.
(DOC)

S2 Fig The plot of sensorimotor task induced tissue density increase versus CBF increase. Difference means task condition minus rest condition. Both measures were extracted from the overlapped suprathreshold sMRI-ATC regions and CBF regions shown in Fig 2A and 2B. Significant correlation (r = 0.66, p = 0.0017) was found between the task-induced changes of the two measures.
(DOC)
