# Supplementary material for: Short-term apparent brain tissue changes are contributed by cerebral blood flow alterations
Source: PLoS One. 2017 Aug 18;12(8):e0182182. doi: 10.1371/journal.pone.0182182 (PMC5562307; doi:10.1371/journal.pone.0182182)
Supplement: S1 Fig — Difference means pre-caffeine minus post-caffeine. Both measures were extracted from the suprathreshold sMRI-ATC regions shown in Fig 1B. No significant correlation between the caffeine-induced changes of the two measures. (DOC) [file pone.0182182.s001.doc]

Supplementary materials

Short-term apparent brain tissue changes are contributed by cerebral blood flow alterations

Qiu Ge^1^, Wei Peng^1^, Jian Zhang^2^, Xuchu Weng^1^, Yong Zhang^3^, Thomas Liu^4^, Yu-Feng Zang^1^, Ze Wang^1,5*^

^1^ Center for Cognition and Brain Disorders, Department of Psychology, Hangzhou Normal University, Hangzhou, China ^2^Department of Physics, Hangzhou Normal University, Hangzhou, China, ^3^GE Healthcare Beijing, China ^4^Department of Radiology, University of California San Diego, San Diego, USA, ^5^Department of Radiology, Lewis Katz School of Medicine, Temple University, Philadelphia, USA


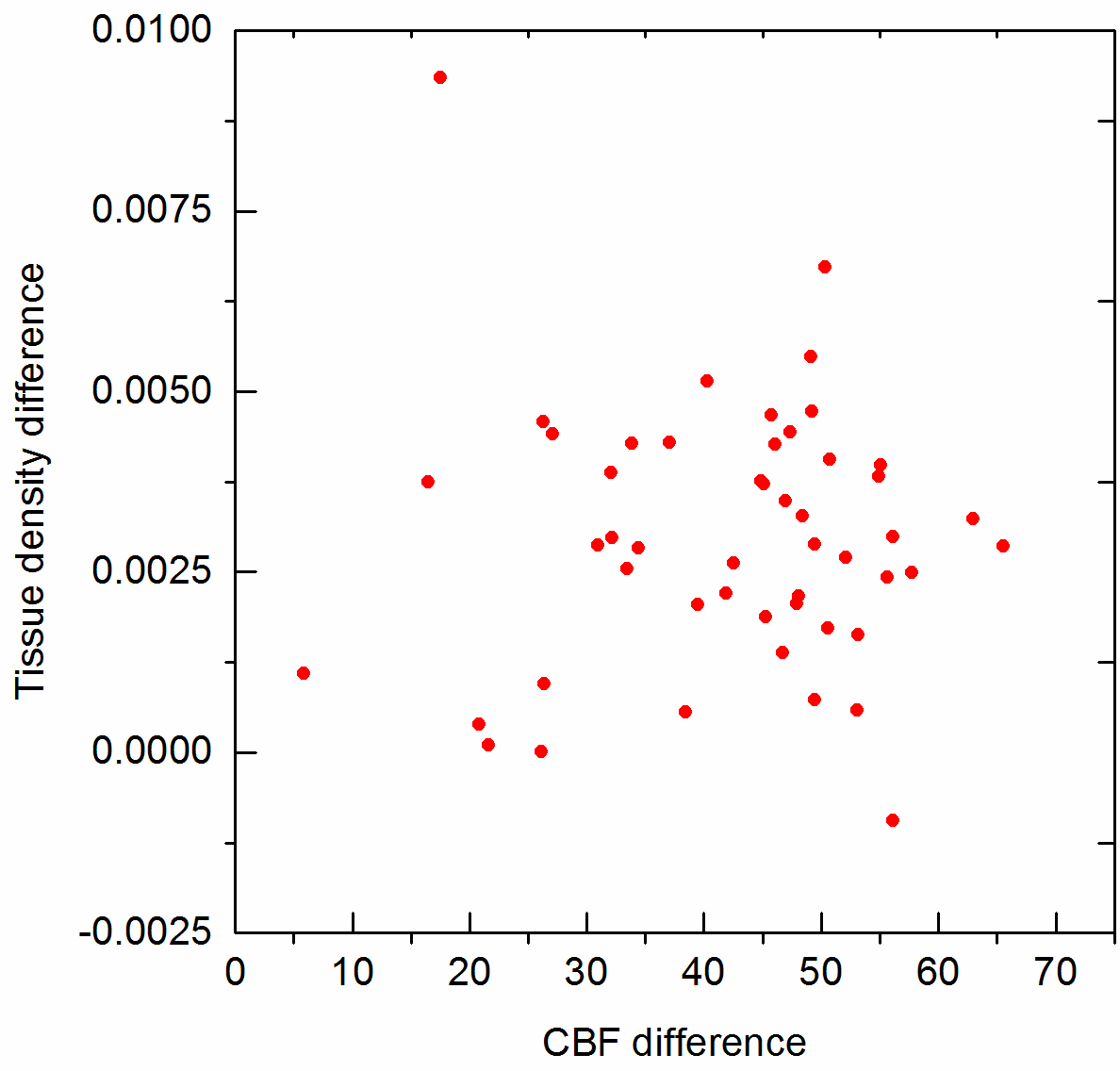


S1 Fig. The plot of caffeine induced tissue density reduction versus CBF reduction. Difference means pre-caffeine minus post-caffeine. Both measures were extracted from the suprathreshold sMRI-ATC regions shown in Fig. 1B. No significant correlation between the caffeine-induced changes of the two measures.
