# Supplementary material for: Short-term apparent brain tissue changes are contributed by cerebral blood flow alterations
Source: PLoS One. 2017 Aug 18;12(8):e0182182. doi: 10.1371/journal.pone.0182182 (PMC5562307; doi:10.1371/journal.pone.0182182)
Supplement: S2 Fig — Difference means task condition minus rest condition. Both measures were extracted from the overlapped suprathreshold sMRI-ATC regions and CBF regions shown in Fig 2A and 2B. Significant correlation (r = 0.66, p = 0.0017) was found between the task-induced changes of the two measures. (DOC) [file pone.0182182.s002.doc]

Supplementary materials

Short-term apparent brain tissue changes are contributed by cerebral blood flow alterations

Qiu Ge^1^, Wei Peng^1^, Jian Zhang^2^, Xuchu Weng^1^, Yong Zhang^3^, Thomas Liu^4^, Yu-Feng Zang^1^, Ze Wang^1,5*^

^1^ Center for Cognition and Brain Disorders, Department of Psychology, Hangzhou Normal University, Hangzhou, China ^2^Department of Physics, Hangzhou Normal University, Hangzhou, China, ^3^GE Healthcare Beijing, China ^4^Department of Radiology, University of California San Diego, San Diego, USA, ^5^Department of Radiology, Lewis Katz School of Medicine, Temple University, Philadelphia, USA


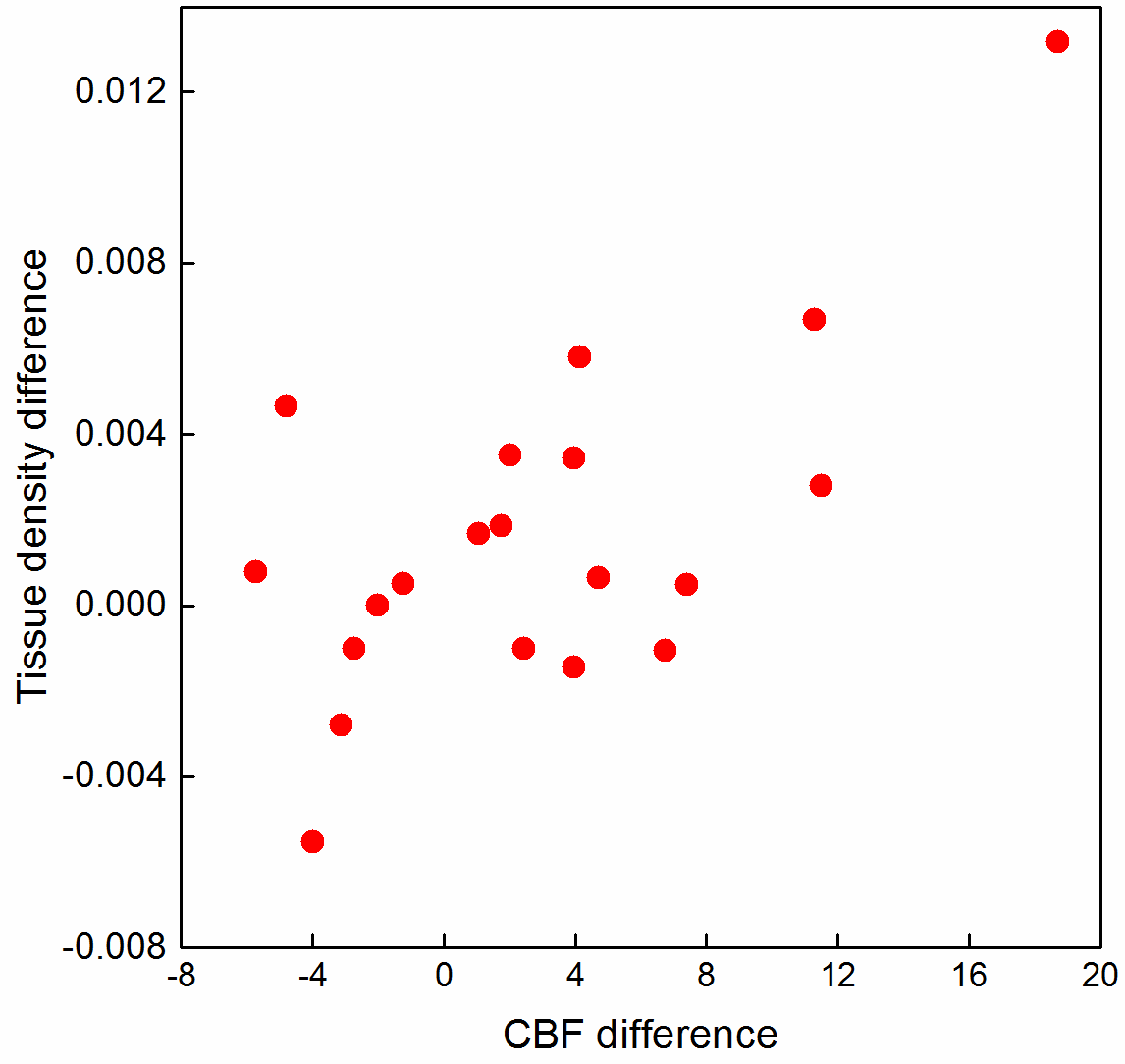


S2 Fig. The plot of sensorimotor task induced tissue density increase versus CBF increase. Difference means task condition minus rest condition. Both measures were extracted from the overlapped suprathreshold sMRI-ATC regions and CBF regions shown in Fig. 2A and 2B. Significant correlation (r=0.66, p=0.0017) was found between the task-induced changes of the two measures.
